# Machine learning analysis for detecting late recurrence and loss to follow‐up after renal cell carcinoma surgery

**DOI:** 10.1002/bco2.425

**Published:** 2024-09-02

**Authors:** Kodai Sato, Tomokazu Sazuka, Takayuki Arai, Hiroaki Sato, Manato Kanesaka, Keisuke Ando, Shinpei Saito, Sangjon Pae, Yasutaka Yamada, Yusuke Imamura, Shinichi Sakamoto, Tomohiko Ichikawa

**Affiliations:** ^1^ Department of Urology, Graduate School of Medicine Chiba University Chiba Japan

**Keywords:** late recurrence, loss to follow‐up, machine learning, random survival forests, renal cell carcinoma

## Abstract

**Objectives:**

Renal cell carcinoma (RCC) is shown to have a tendency for late recurrence, occurring 5 or more years after curative surgery. Imaging diagnosis is required for follow‐up, and there is no definitive answer as to how long this should continue. Some patients discontinue follow‐up visits at their own discretion. How best to predict late recurrence and loss to follow‐up (LF) remains unclear.

**Patients and methods:**

This study targeted patients diagnosed with non‐metastatic RCC who underwent either radical or partial nephrectomy at Chiba University Hospital between 1988 and 2021. Follow‐up for patients with RCC is typically lifelong. We used random survival forests (RSFs), a machine learning‐based survival analysis method, to predict late recurrence and LF. For verification of prediction accuracy, we applied the time‐dependent area under the receiver operating characteristic curve (t‐AUC). To analyse the risks of late recurrence and LF, SurvSHAP(t) and partial dependence plots were used.

**Results:**

We analysed 1051 cases in this study. Median follow‐up was 58.5 (range: 0–376) months. The predictive accuracy of recurrence using RSF was t‐AUC 0.806, 0.761, 0.674 and 0.566 at 60, 120, 180 and 240 months postoperatively, respectively. The recurrence risk impact showed a time‐dependent increase up to approximately 50 months postoperatively. Beyond 50 months, there were no distinct risk factors characteristic of late recurrence. The predictive accuracy of LF using RSF was t‐AUC 0.542, 0.699, 0.685, 0.628 and 0.674 at 60, 120, 180, 240 and 300 months postoperatively, respectively. The risk of LF increased with advancing age beyond 70 years.

**Conclusion:**

It is difficult to identify factors that predict late recurrence. For long‐term follow‐up observation, it is essential to pay particular attention to patients with RCC aged 70 years and above. Establishing frameworks to facilitate collaboration with local hospitals near patients' residences and providing care within the community is necessary.

## 1 INTRODUCTION

Renal cell carcinoma (RCC) accounts for approximately 2% of malignant tumours worldwide, with around 420 000 new diagnoses and approximately 180 000 deaths annually.^1^ Among urological cancers, RCC ranks as the third most common after prostate and bladder cancers.^1^ In recent years, there has been an increasing trend in RCC cases owing to lifestyle changes, coupled with advancements in imaging diagnostics and the dissemination of screenings, leading to an increased diagnosis of early‐stage localised RCC.^2^ Treatment modalities for early‐stage localised RCC include partial nephrectomy or radical nephrectomy performed via open, laparoscopic or robot‐assisted surgery.^3^, ^4^ However, recurrence occurs in 7–30% of patients within 5‐year post‐curative surgery.^5^ Furthermore, RCC has been shown to have a tendency for late recurrence, occurring 5 or more years after curative surgery.^5^, ^6^, ^7^ Therefore, lifelong follow‐up of patients after RCC surgery is deemed crucial.^8^ The development of molecular targeted therapies and immune checkpoint inhibitors has significantly improved the prognosis of patients with recurrent RCC.^9^, ^10^, ^11^, ^12^, ^13^, ^14^, ^15^, ^16^ As a result, the importance of reliable follow‐up for the early detection and treatment of recurrent cases has increased. Therefore, it is imperative to ensure thorough follow‐up for patients at risk of late recurrence. However, evidence for predicting late recurrence post‐RCC surgery remains scarce, necessitating long‐term follow‐up of all cases, which may impose burdens on both patients and healthcare providers. Providing evidence to enable the selective long‐term follow‐up of high‐risk patients by predicting late recurrence and identifying risk factors remains a challenge.

Another challenge during long‐term follow‐up is loss to follow‐up (LF), where patients discontinue follow‐up visits at their own discretion, rendering further follow‐up impossible. Analysing risk factors for LF can potentially aid in careful counselling of such patients to avoid LF.

In this study, we aimed to predict late recurrence and LF in RCC and analyse risk factors to facilitate the avoidance of LF in long‐term follow‐up of kidney cancer.

## 2 PATIENTS AND METHODS

### 2.1 Study design

In this retrospective study, we collected information from patients with RCC treated surgically at Chiba University Hospital in Japan, based on electronic medical records. This study was approved by the Ethics Committee of Chiba University School of Medicine (Ethical Approval Number: 2554). The research was conducted in compliance with the principles outlined in the Declaration of Helsinki, Ethical Principles for Medical Research Involving Human Subjects (as amended in Fortaleza, October 2013). In this study, an opt‐out approach was adopted by the ethics committee for obtaining patient consent. This decision was based on the observational nature of the research, utilising only non‐intrusive and non‐interventional clinical information, thus ensuring no direct impact or intervention on the patients.

### 2.2 Patients

This study targeted patients diagnosed with non‐metastatic RCC who underwent either radical or partial nephrectomy at Chiba University Hospital between 1988 and May 2021. At our institution, follow‐up for patients with RCC is typically lifelong unless the patient becomes unable to visit the hospital owing to difficulties with outpatient visits. Patient information, including age, sex, date of birth, date of surgery, type of surgery (radical or partial nephrectomy), surgical side, surgical approach (open, laparoscopic, robotic), stage, clear cell carcinoma status, pathological nuclear grade using the Fuhrman grading system or the World Health Organization/International Society of Urologic Pathologists grading system, residence, presence of recurrence, time to recurrence, presence of LF and duration until LF, was extracted from the electronic medical records.

### 2.3 Definition of LF

In this study, we defined LF according to our previous research.^17^ LF was defined as cases where it is impossible to retrospectively trace the reasons for the patient's discontinuation of hospital visits, in other words, cases where patients interrupt their hospital visits themselves. Therefore, we did not define cases as untraceable if the discontinuation of hospital visits occurred owing to physician discretion or patient's request, transfer to another hospital or cessation of outpatient visits. In cases where the patient died, the data were censored on the date of death. Furthermore, if patients were referred to another hospital, the data were censored on the date of referral. Additionally, if visits ended after discussions between the attending physician and the patient, the data were censored on the last visit date. In such cases, cancer recurrence or patient survival is not an issue.

### 2.4 Machine learning analysis for late recurrence and LF prediction

We used random survival forests (RSFs), a machine learning‐based survival analysis method, to predict late recurrence and LF.^18^ RSF is an ensemble tree method that uses machine learning to analyse right‐censored survival data. RSF calculates the cumulative hazard function using explanatory variables, occurrence of events and time to events as the supervised data. Because RSF is a supervised learning method, the collected patient data were divided into training (70%) and testing (30%) cohorts, with the training cohort used to build the prediction model. A hold out method was used to select this cohort, and no intentional grouping was performed. The testing cohort was used for accuracy validation. RSF can undergo external validation by adopting external datasets as a testing cohort. This means that LF can be predicted in new data, and the model we created can be implemented in clinical practice. RSF was developed using scikit‐learn version 1.2.1 and Python version 3.10.11.

### 2.5 Validation of predictive accuracy

For the verification of prediction accuracy, we used the time‐dependent area under the receiver operating characteristic curve (t‐AUC).^19^ In survival analysis, global discrimination indices such as Harrell's C‐index may not always be sufficient. The t‐AUC was adopted because it allows tracking of changes in prediction accuracy over time, making the results visually comprehensible. The t‐AUC was computed using the scikit‐survival module version 0.20.0 within the scikit‐learn library.

### 2.6 Risk factor analysis

To analyse the risks of late recurrence and LF, SurvSHAP(t) and the partial dependence plot (PDP) were used.^20^, ^21^ SurvSHAP(t) enables depiction of the relationship between the time elapsed since RCC surgery and the impact of risk factors causing events over time. In real‐world scenarios, the impact of risk factors on event occurrence is not uniform over time, which traditional statistical methods fail to represent. SurvSHAP(t) was adopted for the analysis of risk factors for the late recurrence of kidney cancer because it can express the relationship between elapsed time and the impact of risk factors. The PDP is an intuitive method for understanding how the magnitude of each factor affects the risk, and it allows for the analysis of whether changes in the values of each factor related to late recurrence or LF in kidney cancer increase or decrease the risk.

## 3 RESULTS

### 3.1 Patient characteristics

A total of 1176 cases of RCC surgeries were performed at Chiba University Hospital from 1988 to May 2021. Among these, 1051 cases were analysed, after excluding cases with metastatic RCC and those lacking documentation of the recurrence‐free survival period. Patient characteristics are summarised in Table 1. The median age at the time of surgery was 63 year (range: 16–91), and the median follow‐up period was 58.5 months (range: 0–376). Male patients accounted for 72.4% of the cohort, and female patients accounted for 27.6%. Regarding the comparison between training and testing data, a statistically significant difference (*p* = 0.03) was observed in the proportion of nuclear grade; no statistically significant differences were found in age, follow‐up period, sex distribution, presence of total nephrectomy, surgical approach, laterality, stage, pathological type, recurrence rate or LF rate. There were no discernible differences in trends among these variables.

**TABLE 1:** Patient characteristics.

Baseline characteristic	All patients(*n* = 1051)	Training cohort(*n* = 736)	Testing cohort(*n* = 315)	*p* value
Age at surgery, year (range)	63(16–91)	63(22–91)	63(16–86)	0.766
Follow‐up duration, month (range)	58.5(0–376)	57.9(0–361)	59.0(0.5–376)	0.802
Sex, *n*(%)				0.708
Male	761(72.4)	527(71.6)	234(74.3)	
Female	290(27.6)	209(28.4)	81(25.7)	
Extent of resection, *n*(%)				0.655
Total nephrectomy	637(60.6)	452(61.4)	185(58.7)	
Partial nephrectomy	373(35.5)	257(34.9)	116(36.8)	
Unknown	41(3.9)	27(3.7)	14(4.5)	
Procedure, *n*(%)				0.84
Open	330(31.4)	236(32.1)	94(29.8)	
Laparoscopic	441(42.0)	309(42.0)	132(41.9)	
Robot	218(20.7)	149(20.2)	69(21.9	
Unknown	62(5.9)	42(5.7)	20(6.35)	
Surgical side, *n*(%)				0.884
Right	534(50.8)	370(50.3)	164(52.1)	
Left	510(48.5)	361(49.0)	149(47.3)	
Bilateral	7(0.7)	5(0.7)	2(0.6)	
Stage, *n*(%)				0.357
I	831(79.1)	591(80.3)	240(76.2)	
II	70(6.7)	45(6.1)	25(7.9)	
III	141(13.4)	95(12.9)	46(14.6)	
Unknown	9(0.8)	5(0.7)	4(1.3)	
Pathological type, *n*(%)				0.102
Clear cell RCC	888(84.5)	611(83.0)	277(87.9)	
Others	161(15.3)	123(16.7)	38(12.1)	
Unknown	2(0.2)	2(0.3)	0(0.0)	
Nuclear grade, *n*(%)				0.03
Grade 1–2	849(80.8)	584(79.3)	265(84.1)	
Grade 3–4	140(13.3)	111(15.1)	29(9.2)	
Unknown	62(5.9)	41(5.6)	21(6.7)	
Recurrence, *n*(%)	156(14.8)	110(14.9)	46(14.6)	0.925
Duration until recurrence, month (range)	51.5(0–377)	52.0(0–361)	51.3(0.53–377)	0.598
Lost follow, *n*(%)	173(16.5)	130(17.7)	43(13.7)	0.123
Duration until lost follow, month (range)	58.6(0–377)	57.9(0–361)	59.0(0.5–377)	0.776

Abbreviation: RCC, renal cell carcinoma.

### 3.2 Predictive accuracy for late recurrence

**FIGURE 1:**
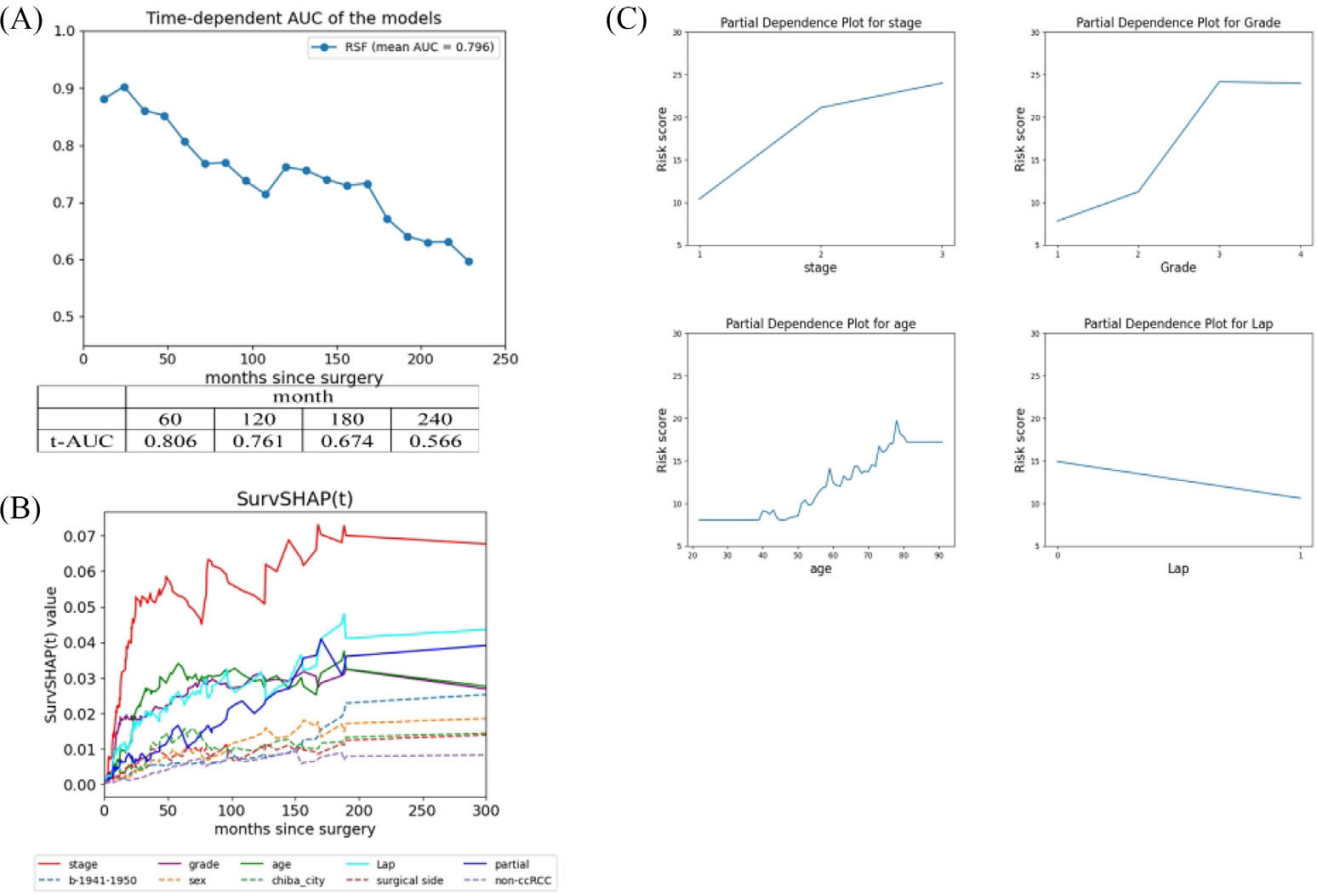
Predicting late recurrence using machine learning. (A) Verification of prediction accuracy using the time‐dependent area under the receiver operating characteristic curve (t‐AUC). (B) Factor analysis according to time course using SurvSHAP(t). (C) Partial dependence plot for leading risk factors.

The predictive accuracy of late recurrence using RSF is illustrated in Figure 1A. The t‐AUC at 60, 120, 180 and 240 months postoperatively was 0.806, 0.761, 0.674 and 0.566, respectively. We observed that the predictive accuracy of RSF decreased over time, making it challenging to anticipate late recurrence.

### 3.3 Risk factors for late recurrence

The analysis results of time‐dependent risk factors for late recurrence using SurvSHAP(t) are presented in Figure 1B. We analysed factors with high variable importance, identified using RSF (Table S1). An explanation of the abbreviations in Figure 1B is provided in Table S2. According to the figure, stage was the most influential risk factor for recurrence, with its impact showing a time‐dependent increase until approximately 50 months postoperatively. Following stage, we identified grade, age and laparoscopic surgery as risk factors. These exhibited similar trends, with their impact as risk factors showing time‐dependent increases until approximately 50 months postoperatively. However, beyond 50 months, the contribution strength of any risk factor plateaued, and there were no distinct risk factors characteristic of late recurrence.

Figure 1C illustrates the relationship between the leading risk factors identified using the PDP and recurrence risk scores. A higher risk score indicates a greater impact of the factor on recurrence. We found a significant difference between stages 1 and 2, with Stage 3 showing the highest recurrence risk. Grades 3 and above posed a high recurrence risk, with little difference between grades 3 and 4. Age was positively correlated with a higher recurrence risk, indicating increased risk with older age. Patients undergoing laparoscopic surgery showed a slightly lower risk of recurrence.

### 3.4 Predictive accuracy for LF

**FIGURE 2:**
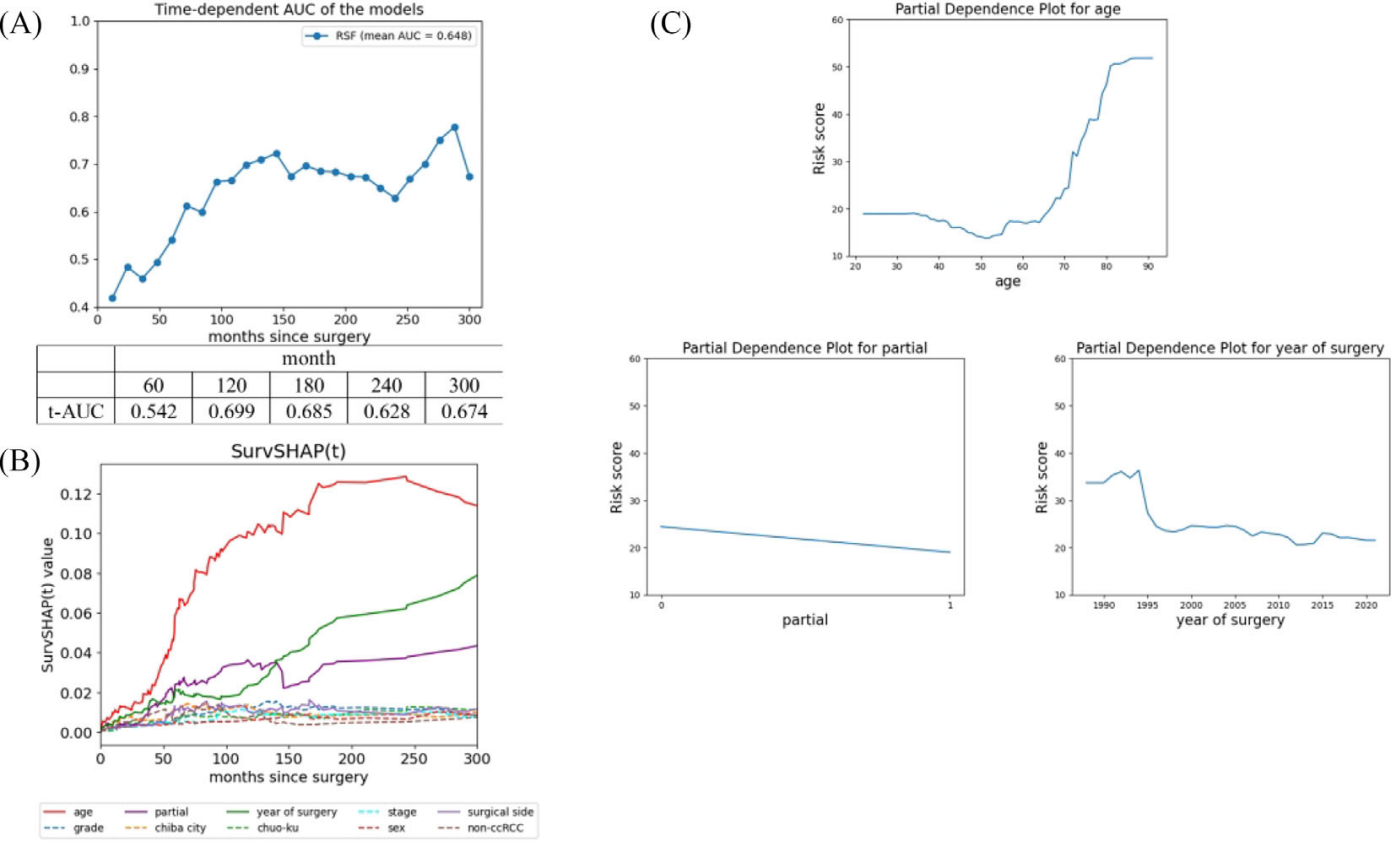
Predicting loss to follow‐up using machine learning. (A) Verification of prediction accuracy using the time‐dependent area under the receiver operating characteristic curve (t‐AUC). (B) Factor analysis according to time course using SurvSHAP(t). (C) Partial dependence plot for leading risk factors.

The predictive accuracy of LF using RSF is illustrated in Figure 2A. The t‐AUC at 60, 120, 180, 240 and 300 months postoperatively was 0.542, 0.699, 0.685, 0.628 and 0.674, respectively. Predicting LF in the early postoperative period was found to be challenging. However, compared with the early postoperative period, an improvement in LF predictive accuracy was observed beyond 10 years postoperatively.

### 3.5 Risk factors for LF

The analysis results of time‐dependent risk factors for LF using SurvSHAP(t) are depicted in Figure 2B. An analysis was conducted on factors with high variable importance identified using RSF (Table S3). An explanation of the abbreviations in Figure 2B is provided in Table S4. It was shown that the impact of age on LF strengthens over time. Subsequently, partial nephrectomy and year of surgery were found to have a significant impact on LF. The impact of other factors on LF was minimal, indicating their relatively small contribution as risk factors for LF.

The results of the PDP for factors that had a strong impact on SurvSHAP(t) are shown in Figure 2C. Individuals aged 70 years or younger had a lower risk of LF, with the LF risk increasing with advancing age beyond 70 years. Patients who underwent partial nephrectomy showed slightly lower risk. Patients who underwent surgery in the 1990s exhibited a higher risk of LF.

## 4 DISCUSSION

The findings of this study confirm that predicting late recurrence of RCC is challenging; however, we found a higher risk of LF in patients aged 70 years and above. By carefully monitoring such patients, it may be possible to avoid LF and provide appropriate treatment for late recurrence of RCC.

The recurrence of RCC following local surgical therapy is reported to occur in approximately 7–30% of cases within 5 years.^5^ Furthermore, there is a trend indicating late recurrence of RCC occurring after 5 years, suggesting the need for lifelong follow‐up.^22^, ^23^ To successfully conduct lifelong follow‐up, it is imperative to educate patients to avoid discontinuation of outpatient visits. Predicting late recurrence, conveying the risk to patients at high risk and maintaining motivation to ensure reliable follow‐up is one approach. However, evidence regarding the prediction of late recurrence remains scarce. Whereas nomograms have been developed to predict recurrence after local treatment of RCC and evidence has been reported for predicting early recurrence, the median observation period in previous studies was 32 months, with the longest observation period being 120 months; moreover, evidence for recurrence prediction exceeding 10 years is limited.^24^ In our study, the median observation period was 52 months, and the longest observation period was 377 months, representing a novel approach to predicting late recurrence based on data from long‐term follow‐up. In this study, the predictive accuracy for late recurrence following RCC surgery decreased with postoperative follow‐up time. Additionally, we conducted machine learning analysis to identify characteristic risk factors for late recurrence, but no clear trends were identified. These findings suggest the difficulty in predicting late recurrence of RCC. Considering these results, we believe that rather than aiming for selective follow‐up of patients with RCC at high risk of late recurrence, it is crucial to take measures to avoid LF.

Regarding the prevention of LF, our prior investigation revealed a heightened risk of LF among elderly adults, a conclusion further supported by our machine learning analysis.^17^ In this study, it is intriguing to note that the predictive accuracy of LF increased over time, particularly between 5 and 10 years postoperatively. The rising predictive accuracy suggests a significant impact of predictive factors, implying a trend towards LF among individuals aged 70 years and above over 5–10 years. It is crucial to prioritise follow‐up strategies to prevent LF among elderly individuals. Factors contributing to LF in high‐risk elderly patients include a decline in activities of daily living (ADL), reduced motivation to attend medical appointments due to ageing, difficulty in commuting due to loss of transportation and forgetting to attend appointments due to the absence of family members who encourage visits. For elderly patients, commuting to high‐volume hospitals where surgeries were performed may involve long distances and lengthy waiting times, which can reduce their motivation to attend follow‐ups. To prevent LF caused by such reasons, we believe that conducting follow‐ups at hospitals closer to the patient's home and establishing a system to remind patients of their appointments would be effective. It is crucial to create an environment where smooth referrals can be made from high‐volume facilities to hospitals near the patient's residence and to actively introduce home nursing services to observe and encourage patient visits. We consider the establishment of such regional collaboration systems to be an effective method to prevent LF. With increasing adoption of minimally invasive techniques such as laparoscopic and robot‐assisted surgeries, the number of elderly patients undergoing RCC surgery is expected to rise.^25^, ^26^ Hence, the need for such regional collaborations is expected to further increase.

This study has several limitations that should be considered. First, this was a retrospective cohort study, including data from more than 30 years ago, which limits the factors available for analysis. Analysing factors that may include crucial information, such as blood test data, could potentially yield new insights. Second, the cohort was from a single institution in Japan. The prognosis of RCC has been reported to vary by race, and analyses involving other racial groups may show trends that differ from those in this study.^27^ Third, there was no predefined follow‐up protocol, and cases may have been discontinued from follow‐up based on the discretion of the attending physician. Analysing such cases may reveal trends in new risk factors. Fourth, LF may be related not only to age but also to other factors such as the presence of cohabitating family members and medical history. However, in this study, it was challenging to collect such data, and thus, an analysis could not be performed. Future additional analyses and data collection should focus on these factors.

In conclusion, even when using machine learning analysis, it remains difficult to identify factors that predict late recurrence. To avoid LF among patients with RCC, it is essential to pay particular attention to long‐term follow‐up of individuals aged 70 years and above. Establishing frameworks that facilitate collaboration with local hospitals near patients' residences and providing care within the community is necessary. We consider this approach crucial to maintaining patient adherence to follow‐up appointments.

## AUTHOR CONTRIBUTIONS

K.S. contributed to analysing data, drawing tables, preparing figures and writing; T.S. contributed to collecting data, analysing data, collecting the bibliography, drawing tables, preparing figures and writing; T.A., H.S., M. K, K. A, S. S, S. P, Y.Y., Y.I. and S.S. contributed to collecting data; T.S. and T.I. contributed to the supervision of all activities. All authors have read and agreed to the published version of the manuscript.

## CONFLICT OF INTEREST STATEMENT

All authors declare that there are no conflicts of interest.

## Supporting information


**Table S1.** Factors for late recurrence with high variable importance, identified using random survival forests.

**Table S2.** Abbreviations of factors for late recurrence.

**Table S3.** Factors for loss to follow‐up with high variable importance, identified using random survival forests.

**Table S4.** Abbreviations of factors for loss to follow‐up.
